# A Simultaneous Pipe-Attribute and PIG-Pose Estimation (SPPE) Using 3-D Point Cloud in Compressible Gas Pipelines

**DOI:** 10.3390/s23031196

**Published:** 2023-01-20

**Authors:** Hoa-Hung Nguyen, Jae-Hyun Park, Han-You Jeong

**Affiliations:** 1 Robotics Institute of Non-Destructive In-Line Inspection (RiNDi), Pusan National University, Busan 46241, Republic of Korea; 2 Department of Electrical Engineering, Pusan National University, Busan 46241, Republic of Korea

**Keywords:** in-line inspection, pipeline inspection gauge (PIG), PIG pose, pipe attributes

## Abstract

An accurate estimation of pipe attributes, pose of pipeline inspection gauge (PIG), and downstream pipeline topology is essential for successful in-line inspection (ILI) of underground compressible gas pipelines. Taking a 3D point cloud of light detection and ranging (LiDAR) or time-of-flight (ToF) camera as the input, in this paper, we present the simultaneous pipe-attribute and PIG-pose estimation (SPPE) approach that estimates the optimal pipe-attribute and PIG-pose parameters to transform a 3D point cloud onto the inner pipe wall surface: major- and minor-axis lengths, roll, pitch, and yaw angles, and 2D deviation from the center of the pipe. Since the 3D point cloud has all spatial information of the inner pipe wall measurements, this estimation problem can be modeled by an optimal transformation matrix estimation problem from a PIG sensor frame to the global pipe frame. The basic idea of our SPPE approach is to decompose this transformation into two sub-transformations: The first transformation is formulated as a non-linear optimization problem whose solution is iteratively updated by the Levenberg–Marquardt algorithm (LMA). The second transformation utilizes the gravity vector to calculate the ovality angle between the geometric and navigation pipe frames. The extensive simulation results from our PIG simulator based on the robot operating system (ROS) platform demonstrate that the proposed SPPE can estimate the pipe attributes and PIG pose with excellent accuracy and is also applicable to real-time and post-processing non-destructive testing (NDT) applications thanks to its high computational efficiency.

## 1. Introduction

Pipelines are the most economical way to transport large quantities of liquid or gas such as oil, water, sewage, natural gas, and hydrogen gas over a long distance. As a result, pipeline transport occupies 70 % of the crude oil and petroleum supply in the United States and 97 % of the natural gas and oil supply in Canada [1,2]. To assess the pipeline integrity, the operators regularly perform a proactive, non-destructive examination of their pipeline, called the **in-line inspection (ILI)**. In the ILI, a **pipeline inspection gauge (PIG)** moves inside of an operational pipeline and collects sensor data, including magnetic, acoustic, optical, inertial, pressure, etc., to identify and localize corrosion, cracks and other defects that may lead to catastrophic failure [3,4,5]. After ILI, these sensor data are post-processed to assess the severity of defects and accurately locate them without digging up excessive amount of pipeline.

In this paper, we focus on the ILI of compressible gas pipelines, where a PIG is driven by the differential pressure between the compressed upstream gas and the operating downstream gas. While a PIG moves forward inside the gas pipeline, multiple **non-destructive testing (NDT)** sensors mounted in the circumferential direction of PIG simultaneously sample their signals for successful assessment of pipeline integrity. For example, a geometry PIG senses its caliper arm angle to detect dents and pipeline fixtures, such as weld, bend, tee, valve, etc. [6,7]. On the other hand, a magnetic flux leakage (MFL) PIG samples the magnetic leakage field from the saturated ferromagnetic pipe to estimate the shape and sizing of pipe defects, such as corrosion, pitting, crack, etc. [8,9]. Our prime interest lies in the **exteroceptive sensing** of PIG, which helps to better recognize pipe attributes and PIG pose.

A key challenge in the ILI of gas pipelines is to keep the PIG speed below a threshold to acquire NDT sensor data of sufficient quality to meet the specifications [5]. Although a small amount of differential pressure is enough to propel a PIG in a straight pipe, downstream pipeline restriction, such as a high curvature bend, heavy wall thickness change, or pipeline debris, may slow down or completely stop the PIG [10]. Then, the upstream gas pressure will build up until the differential pressure exceeds the static friction at the restriction point. This differential pressure is usually much higher than the pressure required to reliably propel the PIG, which results in **speed excursions** [10,11,12]. The phenomenon of speed excursion is known to be much more severe in low-pressure, low-flow gas pipelines, because it takes much longer for the downstream gas to catch up with the compressed upstream pressure. For example, it is reported in [10] that a PIG with speed excursion is broken by slamming to a 1.5 D bend at the downstream of the gas pipeline. To mitigate the damage of the PIG and pipeline from speed excursion, PIGs may throttle the amount of bypassing gas flow, adjust the magnetic attraction of the MFL magnetizer, and/or self-propel to escape from the restriction point before the differential pressure becomes too high.

For the success of PIG speed control, it is essential to accurately estimate the pipe attributes, pose of PIG body, and downstream pipeline topology. To aim this, in-pipe robots are usually equipped with exteroceptive sensors. Two major approaches to exteroceptive sensing have received significant attention from academia and industry: **laser image processing** and **visual odometry**. The laser image processing approach irradiates a laser beam pattern on the inner wall of the pipeline and estimates the pipe attributes or robot pose through the mathematical modeling of the reflected light captured at a camera image [13,14,15,16,17,18]. On the other hand, the visual odometry approach extracts visual features from a camera image, and estimates the relative robot pose by associating them with the corresponding features in the subsequent camera images [19,20,21,22].

Although both exteroceptive sensing approaches are suitable for tethered or self-propelled in-pipe robots, they still face three common limitations in the long-distance ILI of compressible gas pipelines: First, the projection of the inner pipe wall surface onto the camera image plane results in an intrinsic loss of depth information that needs to be recovered from either the complex mathematical model of laser image processing or the association of visual features over subsequent frames. Second, their estimation is incomplete in the sense that they require prior information such as pipe radius with an assumption of a perfectly round cross section in [13,14,15,16,19,20,21], which may cause additional estimation errors to the pipes with different nominal thicknesses or high ovality due to deformation. Third, a high variation of PIG speed can significantly deteriorate the estimation quality of the inner wall geometry, either due to a single plane observation of the pipe surface in [13,14,15,16] or an erroneous association of blurred visual features in [19,20,21,22].

Taking into account these limitations, we advocate the use of **3D point cloud** from the light detection and ranging (LiDAR) [23] or time-of-flight (ToF) camera [24] as an exteroceptive sensing data. Compared to the camera image, the prime difference of a 3D point cloud is that it can directly measure the 3D inner pipe wall surface rather than projecting it onto the 2D image plane. In this paper, we present the **simultaneous pipe-attribute and PIG-pose estimation (SPPE)** approach that estimates the optimal seven pipe geometry and PIG pose parameters for the transformation of a 3D point cloud into the inner pipe wall surface: major- and minor-axis lengths, roll, pitch, yaw angles of PIG, and 2D deviation from the center of pipe in the cross section plane. The basic idea of our SPPE approach is to decompose the whole transformation into two sub-transformations: The first transformation uses the Levenberg–Marquardt algorithm (LMA) [25,26,27,28] to convert the 3D point cloud in the PIG sensor frame (PSF) to a point cloud in the geometric pipe frame (GPF), where the semi-major and semi-minor axes of elliptical pipe cross section are mapped to the Y- and Z-axes of the GPF, respectively. Utilizing the gravity vector, the second transformation calculates the ovality angle to rotate the GPF point cloud to obtain the output point cloud in the navigation pipe frame (NPF). The extensive simulation results from our PIG simulator based on the robot operating system (ROS) platform shows that our SPPE approach can estimate the pipe attributes and PIG pose with outstanding accuracy, and is also used for both real-time and post-processing NDT applications thanks to its high computational efficiency.

The remainder of this paper is organized as follows: In Section 2, we briefly summarize the related works on the exteroceptive sensing of in-pipe robots. Section 3 presents our SPPE approach to the estimation of pipe attributes and PIG pose. Then, the numerical results from our ROS-based PIG simulator are presented and discussed in Section 4. Finally, we conclude this paper in Section 5.

## 2. Literature Review and Our Approach

The objective of ILI is to detect defects on the surface of pipe and accurately estimate their location and attributes for successful pipeline integrity management [5]. In compressible gas pipelines, NDT technologies, such as MFL and Eddy Current (EC), are widely used to detect defects and identify their attributes [8,9], while caliper arms are used for the detection of pipeline deformation [6,7]. In addition, the post-processing of above ground markers (AGMs) and multiple PIG sensors, such as inertial measurement unit (IMU) and odometers, can estimate the location of the PIG and pipe defects in a harsh in-pipe environment, where no external electromagnetic signal penetrates inside the pipeline. However, the above PIG sensors are not sufficient to estimate the pipe attributes, PIG pose, and downstream pipeline topology to avoid speed excursion in compressible gas pipelines.

To mitigate this problem, two exteroceptive sensing approaches have been investigated in the literature: laser image processing and visual odometry. The laser image processing approach estimates the robot pose or pipe diameter by analyzing the shape of the reflected laser image patterns projected onto the camera image plane [13,14,15,16,17,18]. In [13], Kim et al. propose a laser system consisting of four point lasers, a hyperbolic mirror, and an omni-directional camera, and present an algorithm that estimates the rotation angles yielding a specific light pattern on the image plane of the omni-directional camera. A conical laser system is also proposed in [14,15,16,17,18], where a conical laser beam is radiated to the inner wall of the downstream pipe and its reflected light on the camera image plane is analyzed for the estimation of the robot pose or pipe attributes. The robot pose is estimated by the matching pose from the feature database in [14], or computed by the non-linear optimization formulations in [15,16]. For the estimation of pipe diameter, Buschinelli et al. present a direct analysis of the laser patterns in the polar coordinate using Cuda parallel computing in [17], whereas Jin et al. propose a regression analysis of laser points in a camera image in [18]. However, the laser image processing approach has two fundamental limitations in detecting pipe attributes and downstream pipeline topology: (1) it is an incomplete estimation of the pipe attribute and robot pose, because one of them is assumed to be given to estimate the other; and (2) since the laser light pattern in the image plane is usually limited to a set of points in a 2D plane, it is hard to detect the details of 3D features in the downstream pipeline topology, such as bend and tee.

On the other hand, the visual odometry approach extracts the keypoints that commonly exist in multiple camera images, and estimates the robot pose by formulating the pixel differences of these keypoints as the relative differences in the robot poses [19,20,21,22]. Hansen et al. present two monocular visual odometry algorithms to estimate the robot pose in [19]. In [20], they also present a new camera calibration scheme, a sparse bundle-adjustment framework, and the use of a structured lighting system to improve visual odometry and mapping accuracy. Based on the images of a monocular camera, Kagami et al. propose an incremental Structure-from-Motion (SfM) scheme that incorporates the prior constraints into bundle adjustment in order to restore the 3D shape of the whole pipeline [21]. In addition, the influence of the concentrated distribution of 2D visual features in the circumferential direction on the pose estimation of the robot is systematically analyzed in [22]. However, in the visual odometry approach, an incorrect association of visual features between two image frames may result in catastrophic consequences.

**Figure 1:**
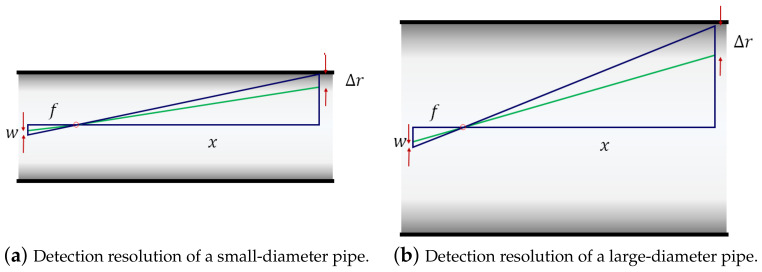
Detection resolution (Δr) of pipe diameter.

Furthermore, both exteroceptive sensing approaches face a few common limitations in their application to the ILI of large-diameter gas pipelines. First, the projection of the inner pipe wall surface onto the camera image plane results in an intrinsic loss of depth information that can be hardly recoverable from the mathematical modeling of laser image processing or the association of visual features over subsequent frames. To overcome this limitation, the approaches in [13,14,15,16,19,20,21] require prior information such as pipe diameter, as well as the assumption of a perfectly round pipe cross section. Second, a fish-eye camera with a wide FoV in a large-diameter pipe limits the detection resolution of pipe surface features due to its pixel granularity. Figure 1 shows an example of the resolution of pipe surface detection with different pipe diameters, where the notations *f*, *w*, *x*, and Δr represent the focal length, pixel width, depth, and the resolution of pipe surface detection, respectively. Given the camera parameters, the detection resolution of the pipe surface is represented by Δr=xw/f, which increases with the pipe diameter and camera FoV as shown in Figure 1a,b. Third, a high variation of PIG speed can significantly deteriorate the estimation quality of inner wall geometry due to the irregular spacing between the observed pipe surface planes or erroneous association of blurred visual features.

In this paper, we propose an alternative exteroceptive sensing approach that uses a 3D point cloud of a LiDAR or ToF camera for the ease of detecting pipe attributes, PIG pose, and downstream pipeline topology. Since the -D point cloud retains all spatial information of the inner pipe wall measurements, this approach can avoid the limitation originating from their projection onto a 2D camera image plane, and provide a principled methodology to address a few fundamental issues of pipe-attribute and PIG-pose estimation: first, this estimation problem is modeled by the transformation matrix estimation problem from a 3D point cloud in the PSF to a 3D point cloud in the global NPF. Second, the PIG-pose parameters are clearly distinguished from the pipe-attribute parameters in our problem specification—the former constitutes the rotation and translation matrices, while the latter is used for the objective function of the optimization formulation. Third, our iterative solution based on the LMA can estimate the pipe attributes and PIG pose with excellent accuracy and be used for many real-time and post-processing NDT applications.

## 3. A Simultaneous Pipe-Attribute and PIG-Pose Estimation (SPPE)

In this section, we first formulate the model for the reference frames, and describe the transformation invariance of 3-D point cloud measurement in Section 3.1. Given the input point cloud of the PSF, the objective of our SPPE approach is to find the optimal pipe-attribute and PIG-pose parameters of a transformation by which the output point cloud minimally deviate from the inner pipe wall surface in the NPF. The basic idea of our SPPE approach is to split this transformation into two sub-transformations: The first from the PSF to the GPF in Section 3.2, and the second from the GPF to the NPF in Section 3.3.

### 3.1. Model for Reference Frames and Transformation Invariance

**Figure 2:**
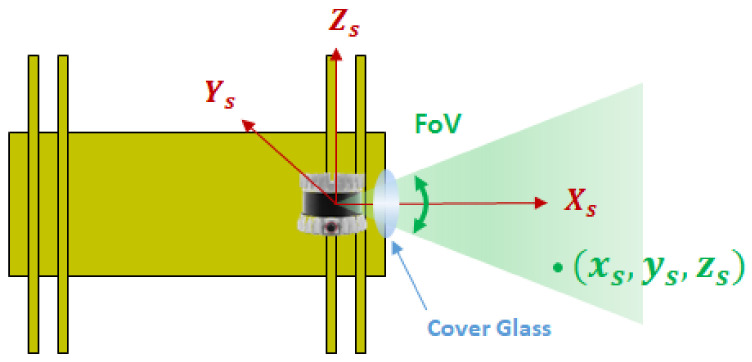
Illustration of PIG sensor frame (PSF).

In this section, we define the reference frames whose origin and orientation are specified by a set of three orthogonal reference axes. Figure 2 shows the PSF whose origin is the center of the 3D ranging sensor, where the *X*-, *Y*-, and *Z*-axes are defined as the front direction (denoted by $X_{s}$), the right direction viewed from the front (denoted by $Y_{s}$), and the upward direction of PIG (denoted by $Z_{s}$), respectively. The 3D ranging sensor periodically generates a 3D point cloud of *m* inner pipe wall measurements, where each point is denoted by $x_{s}$ = ($x_{s}$, $y_{s}$, $z_{s}$). The depth measurement noise of the 3D ranging sensor is assumed to follow a zero-mean Gaussian distribution with standard deviation of σ, i.e., $N(0,\sigma^{2})$.

To protect the 3D ranging sensor from the operating high pressure of gas pipe, it must be placed inside the cover glass, as shown in Figure 2. Usually, a thin coating film is attached to the surface of cover glass to provide better optical transparency to the infrared light from 3D ranging sensor. In this paper, we assume a circular cover glass with field of view (FoV) Ω that has the following two characteristics: (1) it is mechanically strong enough to withstand the differential pressure; and (2) it provides perfect optical transparency for 3D ranging sensor.

Due to the manufacturing deformation and/or stress from underground external forces, the cross section of the pipe is usually represented by an ellipse. To account for this phenomenon, the inner pipe wall is modeled as a cylinder with an elliptical cross section, where *L*, $D_{\max}$ and $D_{\min}$ denote its axial, major-axis, and minor-axis lengths, respectively. Then, the ovality of the inner pipe wall is defined as follows:

(1) $$O\left(\%\right)=2\times \left[\frac{D_{\max}-D_{\min}}{D_{\max}+D_{\min}}\right]\times 100.$$

**Figure 3:**
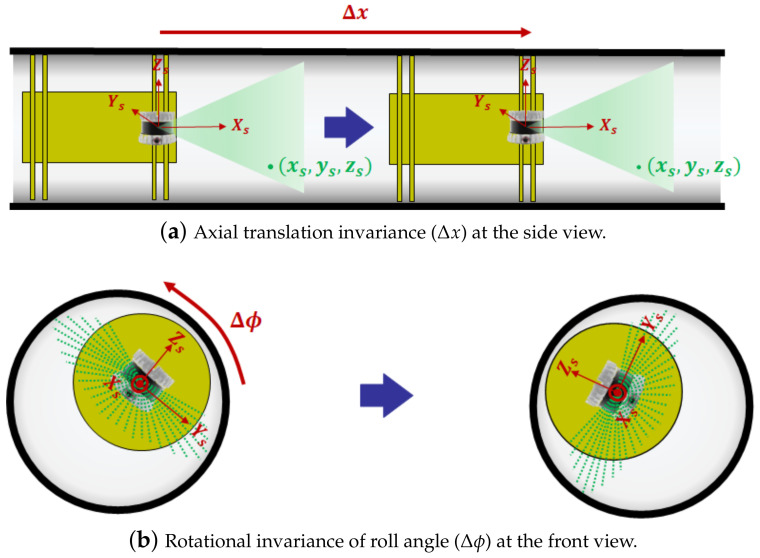
Transformation invariance of 3-D point cloud in a cylindrical pipe.

When pipe length *L* is long enough ($L\gg \Delta x+\max(x_{s})$), the 3D point cloud in the PSF cannot be distinguishable from the point cloud of the following two transformations in a cylindrical pipe: axial translation Δx and rotation of roll angle Δϕ. For example, in Figure 3a, the point clouds of two PIG poses in a cylindrical pipe with a small axial displacement of Δx, while keeping the remaining five degrees-of-freedom (DoF) poses, are indistinguishable from each other because the pipe inner wall geometry observed from both PSFs remains the same. In other words, the 3D ranging sensor is oblivious to the axial displacement Δx of the cylindrical pipe. To overcome this limitation, a fusion of IMU, odometer, and AGM data is used for the estimation of axial displacement [29], which is beyond the scope of this paper. The rotational invariance of roll angle Δϕ in a cylindrical pipe with a perfectly round cross section (O=0) is also shown in Figure 3b. This rotational invariance can result in much higher uncertainties in the roll angle estimate than the pitch and yaw angle estimates for cylindrical pipes with a nearly circular cross section (O≈0), which will be addressed in Section 4.3.

**Figure 4:**
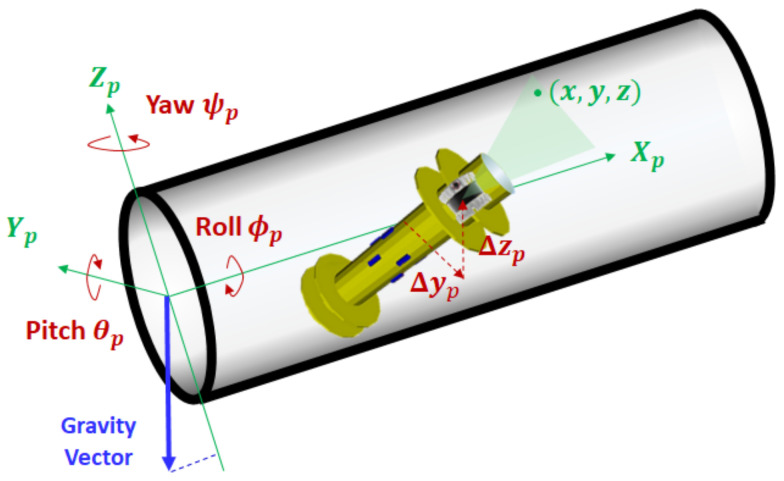
Navigation pipe frame.

Figure 4 shows the definition of the NPF and the corresponding PIG pose, where the origin is the center of the starting pipe section, and the X-axis $X_{p}$ is the axial direction of the cylindrical pipe. For the Z-axis of the NPF, we use an external constant vector that can be directly observable by the IMU sensor: the gravity vector. Then, the Z-axis $Z_{p}$ is defined as the opposite direction to the projected gravity vector onto the starting pipe section. Given this axis definition, we denote the roll, pitch, and yaw angles of PIG in the NPF by $\phi_{p}$, $\theta_{p}$, and $\psi_{p}$, respectively. The 2D deviation from the elliptical center of the cross section plane is also denoted by ($\Delta y_{p}$, $\Delta z_{p}$). Then, our problem can be formulated as follows: Given the input point cloud $x_{s}$ = ($x_{s}$, $y_{s}$, $z_{s}$) of m inner pipe wall measurements in the PSF, our objective is to find the optimal transformation in terms of $\beta_{p}^{*}=\left(D_{\max}^{*},D_{\min}^{*},\phi_{p}^{*},\theta_{p}^{*},\psi_{p}^{*},\Delta y_{p}^{*},\Delta z_{p}^{*}\right)$ by which the output point cloud x = (x, y, z) in the NPF matches well with the inner surface of cylindrical pipe. Here, the major- and minor-axis lengths $D_{\max}^{*}$ and $D_{\min}^{*}$ belong to the pipe attribute, while 5-DoF parameters $\phi_{p}^{*},\theta_{p}^{*},\psi_{p}^{*},\Delta y_{p}^{*}$, and $\Delta z_{p}^{*}$ correspond to the PIG pose.

The basic idea of our SPPE approach is to decompose this transformation into two sub-transformations by devising the GPF as an intermediate reference frame. The introduction of the GPF significantly simplifies the modeling of pipe-attribute and PIG-pose estimation: First, the GPF axes are determined to well match with the geometry of pipe inner wall surface, which enables the pipe cross section to be represented by the canonical ellipse equation. This representation reduces the complexity of the non-linear optimization formulation in the first sub-transformation. Second, it also allows the second sub-transformation to be rotated only in the direction of the roll angle. This rotation angle can be estimated by exploiting the gravity vector of the IMU device. The details of each sub-transformation will be addressed in the following two sections.

### 3.2. Transformation to the Geometric Pipe Frame (GPF)

**Figure 5:**
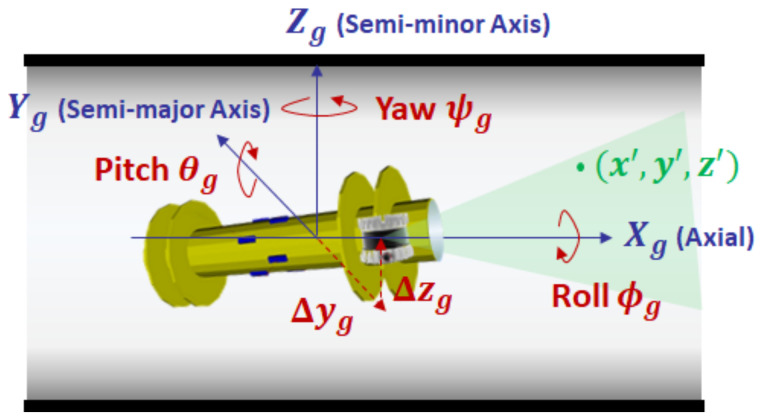
Geometric pipe frame.

In this section, we address the first transformation to convert the input point cloud in the PSF to an intermediate point cloud in the GPF. Figure 5 shows an example of the GPF, where the *X*-, *Y*-, and *Z*-axes are defined as the front axial direction (denoted by $X_{g}$), the right semi-major axis direction viewed from the front (denoted by $Y_{g}$), and the upward semi-minor axis direction of cylindrical pipe (denoted by $Z_{g}$), respectively. We denote the roll, pitch, and yaw angles of the 3D ranging sensor by $\phi_{g}$, $\theta_{g}$, and $\psi_{g}$, respectively. Since the point cloud is invariant with axial translation Δx, we focus on the 2D deviation ($\Delta y_{g}$, $\Delta z_{g}$) from the elliptical center of the cross section plane. Then, an inner pipe surface point $x^{\prime }=$($x^{\prime }$, $y^{\prime }$, $z^{\prime }$) of the GPF can be obtained by the rotation and translation of input point $x_{s}$, as follows:

(2) $$\left[\begin{array}{c} x^{\prime }\\ y^{\prime }\\ z^{\prime }\\ 1 \end{array}\right]=\left[\begin{array}{cc} R_{XYZ}\left(\phi_{g},\theta_{g},\psi_{g}\right) & T_{YZ}\left(\Delta y_{g},\Delta z_{g}\right)\\ 0^{1\times 3} & 1 \end{array}\right]\left[\begin{array}{c} x_{s}\\ y_{s}\\ z_{s}\\ 1 \end{array}\right],$$

where 3D rotation matrix $R_{XYZ}\left(\phi_{g},\theta_{g},\psi_{g}\right)$ can be represented by the multiplication of three rotation matrices, i.e., $R_{XYZ}\left(\phi_{g},\theta_{g},\psi_{g}\right)=R_{Z}\left(\psi_{g}\right)R_{Y}\left(\theta_{g}\right)R_{X}\left(\phi_{g}\right)$, and 2-D translation matrix by $T_{YZ}\left(\Delta y_{g},\Delta z_{g}\right)={\left[0\;\;\Delta y_{g}\;\;\Delta z_{g}\right]}^{⊺}$. When the rotation in pitch angle $\theta_{g}$ is equal to $\pm \frac{\pi }{2}$, the rotation in yaw and roll directions corresponds to the same motion, which is known as the gimbal-lock problem [30]. This problem can be solved by using quaternions or rotation vectors, but they would lead to a much more complex parameter estimation problem than those in (8)–(10). On the other hand, we notice that the absolute pitch angle from PSF to GPF is much less than $\frac{\pi }{2}$ thanks to the fact that PIGs are usually well aligned with the pipeline to shut off the gas flow. As a result, the use of the Euler angles in (2) can still avoid the gimbal-lock problem in the transformation matrix.

Thanks to the mapping of the semi-major and semi-minor axes onto the X- and Y-axes of the GPF, respectively, the equation for the inner surface of the cylindrical pipe is greatly simplified to the canonical ellipse form, as follows:

(3) $$\frac{y^{\prime 2}}{{\left(D_{\max}/2\right)}^{2}}+\frac{z^{\prime 2}}{{\left(D_{\min}/2\right)}^{2}}=1,\;\;\;for\;0\leq x^{\prime }\leq L.$$

In general, transformed point $x^{\prime }$ deviates from the inner pipe wall surface in (3) due to the depth measurement noise of 3D ranging sensors. However, the linearity of the first transformation ($x_{s}\to x^{\prime }$) in (2) allows us to transform a point cloud inside (outside) the pipe surface of the PSF to a point cloud inside (outside) the pipe surface of the GPF.

Given the PSF point cloud $x_{s}$, our first parameter estimation problem is formulated by a non-linear least-square problem that fits the parameterized GPF point cloud $x^{\prime }$ to a mathematical model by minimizing the sum of the squared errors $S(\beta_{g})$ between them, i.e.,

(4) $$\beta_{g}^{*}=\arg\min_{\beta_{g}}S\left(\beta_{g}\right)=\arg\min_{\beta_{g}}\sum_{i=1}^{m}f^{2}\left(x_{i}^{\prime },\beta_{g}\right),$$

where target variable $\beta_{g}$ is the parameters that represent pipe attributes $\left(D_{\max},D_{\min}\right)$ and PIG pose $\left(\phi_{g},\theta_{g},\psi_{g},\Delta y_{g},\Delta z_{g}\right)$ in the GPF, and $f(x_{i}^{\prime },\beta_{g})$ is the cost function to reflect the deviation of transformed point $x_{i}^{\prime }$ from the inner pipe wall surface in (3).

(5) $$f\left(x_{i}^{\prime },\beta_{g}\right)=\frac{y_{i}^{\prime 2}}{{\left(D_{\max}/2\right)}^{2}}+\frac{z_{i}^{\prime 2}}{{\left(D_{\min}/2\right)}^{2}}-1.$$

Since both $y^{\prime }$ and $z^{\prime }$ are trigonometric functions of PIG pose in (2), cost function $f(x_{i}^{\prime },\beta_{g})$ becomes a non-linear function of seven unknown variables $\beta_{g}$.

The optimal solution $\beta_{g}^{*}$ to the non-linear least-square problem can be obtained by the iterative update approach [25,26,27,31,32]. Starting with an arbitrary initial parameter $\beta_{g}^{\left(0\right)}$, this approach iteratively updates the parameter $\beta_{g}^{\left(k+1\right)}=\beta_{g}^{\left(k\right)}+\delta_{k}$ until it converges to an optimal value $\beta_{g}^{*}$, where $\delta_{k}$ is the increment vector. Three iterative update approaches are extensively studied in the literature: the gradient-descent algorithm (GDA) [31], the Gauss-Newton algorithm (GNA) [32], and the LMA [25,26,27].

The GDA iteratively updates the parameters in the steepest-descent direction, i.e.,

(6) $$\beta_{g}^{\left(k+1\right)}=\beta_{g}^{\left(k\right)}-\alpha \nabla S\left(\beta_{g}\right)=\beta_{g}^{\left(k\right)}-\alpha J^{⊺}f\left(x^{\prime },\beta_{g}^{\left(k\right)}\right),$$

where α is the learning rate that determines the length of the step. Function $f\left(x^{\prime },\beta_{g}^{\left(k\right)}\right)$ and m×7 Jacobian matrix J are also given by

(7) $$f\left(x^{\prime },\beta_{g}^{\left(k\right)}\right)={\left[\begin{array}{cccc} f\left(x_{1},\beta_{g}^{\left(k\right)}\right) & f\left(x_{2},\beta_{g}^{\left(k\right)}\right) & \cdots & f\left(x_{m},\beta_{g}^{\left(k\right)}\right) \end{array}\right]}^{⊺},$$

and

(8) $$J=\frac{\partial f\left(x^{\prime },\beta_{g}^{\left(k\right)}\right)}{\partial \beta_{g}^{\left(k\right)}}=\left[\begin{array}{ccccccc} \frac{\partial f}{\partial D_{\max}^{\left(k\right)}} & \frac{\partial f}{\partial D_{\min}^{\left(k\right)}} & \frac{\partial f}{\partial \phi_{g}^{\left(k\right)}} & \frac{\partial f}{\partial \theta_{g}^{\left(k\right)}} & \frac{\partial f}{\partial \psi_{g}^{\left(k\right)}} & \frac{\partial f}{\partial \Delta y_{g}^{\left(k\right)}} & \frac{\partial f}{\partial \Delta z_{g}^{\left(k\right)}} \end{array}\right],$$

respectively. In (6), the GDA moves in the opposite direction of the gradient $\nabla S(\beta_{g})$ at each iteration, eventually reaching a local minimum. However, it is difficult to choose a proper learning rate α: A small learning rate suffers from slow convergence while a large learning rate may lead to the fluctuation or divergence of objective function $S(\beta_{g})$ [31].

On the other hand, the GNA exploits the second-derivative (curvature) information to compute the increment vector [32]:

(9) $$\left(J^{⊺}J\right)\delta_{k}=J^{⊺}f\left(x^{\prime },\beta_{g}^{\left(k\right)}\right).$$

It approximates the objective function $S(\beta_{g})$ locally with quadratic function, and moves to the extreme point of the quadratic function in one step. Although it converges much faster than the GDA for moderate-size problems, the GNA may converge slowly or diverge if the initial parameter $\beta_{g}^{\left(0\right)}$ is far away from the minimum $\beta_{g}^{*}$ or the matrix $J^{⊺}J$ is ill-conditioned.

To mitigate this problem, the LMA adaptively combines the GDA and GNA using the damping factor $\lambda_{k}$, as follows [25,26,27]:

(10) $$\left[J^{⊺}J+\lambda_{k}\;diag\left(J^{⊺}J\right)\right]\delta_{k}=J^{⊺}f\left(x^{\prime },\beta_{g}^{\left(k\right)}\right).$$

The LMA updates its parameter $\beta_{g}^{\left(k\right)}$ like the GDA (with a large value of $\lambda_{k}$) when the it is far from the optimal parameter $\beta_{g}^{*}$, and like the GNA (with a small value of $\lambda_{k}$) when it is near the optimal parameter. More specifically, starting with a large initial value $\lambda_{0}$ and a factor ν>1, the damping factor $\lambda_{k}$ at the *k*-th iteration is adaptively determined, as follows [27]: If damping factor $\lambda_{k}/\nu$ reduces the sum of squared errors $S(\beta_{g})$, damping factor $\lambda_{k+1}$ is set to $\lambda_{k}/\nu$. If $\lambda_{k}$ reduces $S(\beta_{g})$ while $\lambda_{k}/\nu$ increases $S(\beta_{g})$, the damping factor is left unchanged, i.e., $\lambda_{k+1}=\lambda_{k}$. If both of damping factors are worse than the current sum of squared errors, the damping factor is increased by successive multiplication by ν until a better one is found, i.e., $\lambda_{k+1}=\lambda_{k}\nu^{\omega }$ for some positive integer ω. The accuracy of iterative LMA will be addressed in Section 4.2.

### 3.3. Transformation to the Navigation Pipe Frame (NPF)

**Figure 6:**
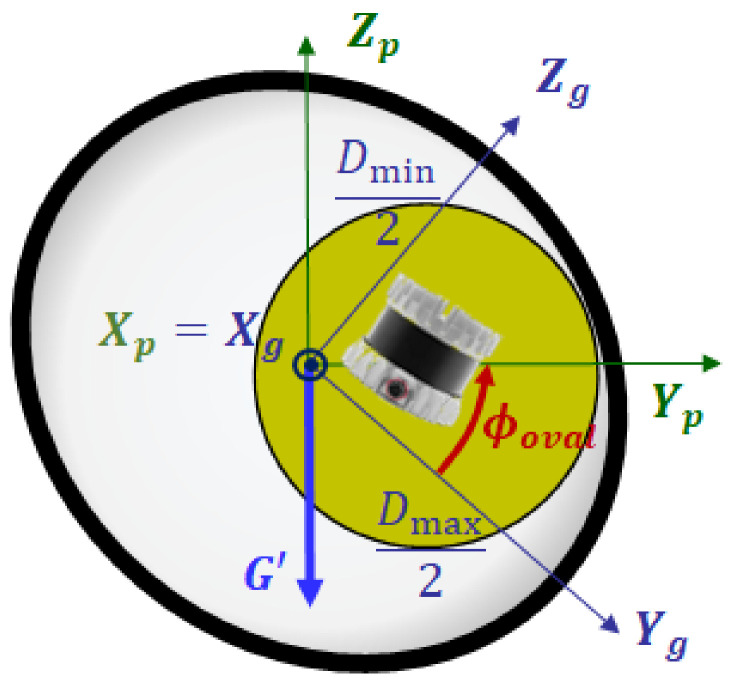
Cross section view of two pipe frames.

In this section, we present the second transformation by which an intermediate point cloud in the GPF is converted to an output point cloud in the NPF. Figure 6 shows the cross section view of these two pipe frames. Since both pipe frames share the same X-axis ($X_{p}=X_{g}$), the transformation from the GPF to the NPF is done by a single rotation in roll direction $\phi_{oval}$, which is called the ovality angle. This angle can be computed by using the gravity vector, as follows: From the relative pose of the 3D ranging sensor to the IMU in the PIG system, we can represent the gravity vector $G_{s}$ in the PSF. The first transformation in (2) and projection to YZ plane of the GPF converts this vector to the projected gravity vector $G^{\prime }=\left(g_{x}^{\prime },g_{y}^{\prime },g_{z}^{\prime }\right)$. Finally, from the roll angle of vector $G^{\prime }$ in Figure 6, the ovality angle $\phi_{oval}$ can be calculated by

(11) $$\phi_{oval}=\frac{\pi }{2}+\arctan\left(\frac{g_{z}^{\prime }}{g_{y}^{\prime }}\right).$$

We notice that, since the second transformation is the rotation in roll angle, the major- and minor-axis lengths remain the same. Given the ovality angle $\phi_{oval}$ and optimal parameters of the first transformation $\beta_{g}^{*}=\left(D_{\max}^{*},D_{\min}^{*},\phi_{g}^{*},\theta_{g}^{*},\psi_{g}^{*},\Delta y_{g}^{*},\Delta z_{g}^{*}\right)$, output point cloud x=(x,y,z) of the NPF can be obtained by

(12) $$\begin{array}{c} \begin{array}{cc} \left[\begin{array}{c} x\\ y\\ z \end{array}\right]\; & =R_{X}\left(\phi_{oval}\right)\left[\begin{array}{c} x^{\prime }\\ y^{\prime }\\ z^{\prime } \end{array}\right]\\ & =R^{p}\left[\begin{array}{c} x_{s}\\ y_{s}\\ z_{s} \end{array}\right]+R_{X}\left(\phi_{oval}\right)\left[\begin{array}{c} 0\\ \Delta y_{g}^{*}\\ \Delta z_{g}^{*} \end{array}\right], \end{array} \end{array}$$

where rotation matrix $R^{p}$ is the multiplication of four rotation matrices:

(13) $$R^{p}=R_{X}\left(\phi_{oval}\right)R_{XYZ}\left(\phi_{g}^{*},\theta_{g}^{*},\psi_{g}^{*}\right)=R_{X}\left(\phi_{oval}\right)R_{Z}\left(\psi_{g}^{*}\right)R_{Y}\left(\theta_{g}^{*}\right)R_{X}\left(\phi_{g}^{*}\right).$$

Denoting the (*i*, *j*) element of 3×3 matrix $R^{p}$ by $r_{ij}^{p}$, the optimal rotation and translation parameters of the whole transformation $\beta_{p}^{*}=\left(D_{\max}^{*},D_{\min}^{*},\phi_{p}^{*},\theta_{p}^{*},\psi_{p}^{*},\Delta y_{p}^{*},\Delta z_{p}^{*}\right)$ are

(14) $$\phi_{p}^{*}=\arctan\left(\frac{r_{32}^{p}}{r_{33}^{p}}\right),\;\theta_{p}^{*}=\arcsin\left(-r_{31}^{p}\right),\;and\;\psi_{p}^{*}=\arctan\left(\frac{r_{21}^{p}}{r_{11}^{p}}\right),$$

and

(15) $$\left[\begin{array}{c} 0\\ \Delta y_{p}^{*}\\ \Delta z_{p}^{*} \end{array}\right]=R_{X}\left(\phi_{oval}\right)\left[\begin{array}{c} 0\\ \Delta y_{g}^{*}\\ \Delta z_{g}^{*} \end{array}\right],$$

respectively.

## 4. Numerical Results

In this section, we validate the accuracy of our SPPE approach based on the simulation results from our ROS-based PIG simulator. We first describe the ROS-based SPPE simulator and simulation environment in Section 4.1. Next, we examine the accuracy of iterative LMA solution to the SPPE problem in Section 4.2. In Section 4.3, we discuss the accuracy of our SPPE in terms of pipe attributes and PIG pose. The robustness of our SPPE to the depth error and LMA input size is finally discussed in Section 4.4.

### 4.1. Ros-Based PIG Simulation

**Figure 7:**
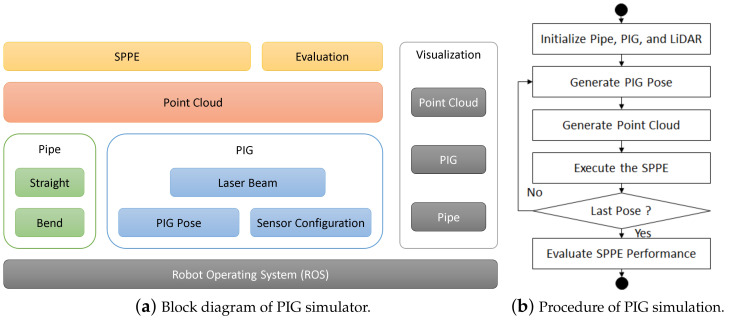
Our PIG simulator.

Based on the Robot Operating System (ROS) [33] framework, a PIG simulator in Figure 7 is developed to generate 3D point cloud of inner pipe wall surface and to evaluate the performance of the proposed SPPE for given pipe attributes and PIG pose. Figure 7a shows the architecture of the PIG simulator consisting of six components: pipe, PIG, point cloud, SPPE, evaluation, and visualization.

The pipe component supports two pipe topologies (straight and bend), where our focus in this paper is on the cylindrical straight pipe with elliptical cross section. Four different pipe diameters (16${}^{\prime \prime }$, 20${}^{\prime \prime }$, 24${}^{\prime \prime }$, and 30${}^{\prime \prime }$) are considered in our PIG simulation, where their nominal thickness is fixed to T=12 mm, the ovality O ranges from zero to two percents, and the ovality angle $\phi_{oval}$ is uniformly distributed over interval ($-180^{\circ }$, $180^{\circ }$). At each configuration, the PIG simulator randomly chooses the value of the pipe attributes including the major- and minor-axis lengths, and ovality angle. Figure 7b shows the overall procedure of PIG simulation. For each pipe configuration, the PIG simulator generates *N* different PIG poses, and for each of them, generates the input point cloud for *m* pipe inner wall measurements. Based on this point cloud, it executes the SPPE to obtain the estimate of pipe attribute and PIG pose. Once all PIG poses are processed, the PIG simulator evaluates the performance of SPPE.

**Figure 8:**
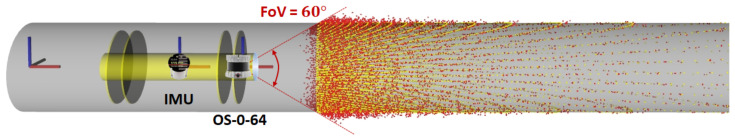
A PIG in a cylindrical pipe.

Figure 8 shows a PIG in an infinitely long cylindrical pipe (L=∞). A PIG is illustrated by a yellow cylinder with two propulsion cups near each end to seal the gas. The PIG component manages the hardware configuration and the pose of PIG body. The sensor configuration module stores the sensor information, such as the number and direction of the beam, the minimum and maximum depths, accuracy, etc. We assume that the Ouster OS-0-64 LiDAR is used for the 3D ranging sensor, where the FoV is $360^{\circ }\times 90^{\circ }$, the angular resolution is 2048×64, and the depth error is σ=0.03 m [23]. It is also assumed that the relative pose of LiDAR to IMU is given by the CAD drawing of PIG. The circular cover glass protects both the IMU and 3D ranging sensor from the high gas pressure inside the pipe, but limits the FoV of LiDAR to $\Omega =60^{\circ }$. However, we believe that the impact of widening the FoV is marginal, because it leads to additional measurement points at close X-axis distance in Figure 8, which has less information about the pose of PIG than distant X-axis points. Given the PIG pose, the laser beam module computes the direction vector $\vec{d}$ ($∥\vec{d}∥=1$) of each laser beam radiating from the LiDAR.

**Figure 9:**
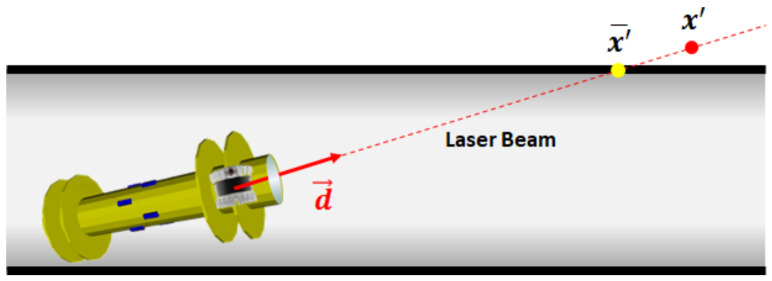
True and measured inner pipe wall points.

The point cloud component is responsible for generating the point cloud of the inner pipe wall measurements. The yellow and red points on the downstream inner pipe wall in Figure 8 represent the true and measured point clouds of LiDAR, respectively. Due to the incident angle, the impact of LiDAR depth error decreases with the axial distance. To account for the attenuation of reflected lights, our PIG simulator also limits the maximum axial distance of measurement to 6 m. Figure 9 shows an example of the true and measured inner pipe wall points. The true point ${\bar{x}}^{\prime }$ between the laser beam and inner pipe wall surface is obtained by solving the following two equations:

(16) $$x^{\prime }=x_{PIG}^{\prime }+t\;\cdot \;\vec{d},\;and\;\frac{y^{\prime 2}}{{\left(D_{\max}/2\right)}^{2}}+\frac{z^{\prime 2}}{{\left(D_{\min}/2\right)}^{2}}=1,$$

where $x^{\prime }=\left(x^{\prime },y^{\prime },z^{\prime }\right)$, *t* is a non-negative real number, and $x_{PIG}^{\prime }$ is the current position of 3-D ranging sensor in the GPF. The measured inner pipe wall point $x^{\prime }$ is obtained by adding a Gaussian depth error to true point ${\bar{x}}^{\prime }$.

The SPPE and the evaluation components are responsible for the execution and performance evaluation of our SPPE, respectively. Finally, the visualization component is responsible for displaying the PIG simulation in the RVIZ, a 3D visualization tool of ROS.

Table 1 shows the simulation parameters to obtain our numerical results. Unless otherwise stated, the numerical results in the following sections are obtained from *N* = 100 random LiDAR poses uniformly distributed over roll angle $\phi_{p}\in \left[-180,180\right)$, pitch and yaw angles $\theta_{p},\psi_{p}\in \left[-5,5\right]$, and displacement from pipe center $\Delta y_{p},\Delta z_{p}\in \left[-50,50\right]$.

### 4.2. Accuracy of Iterative LMA Solutions

**Figure 10:**
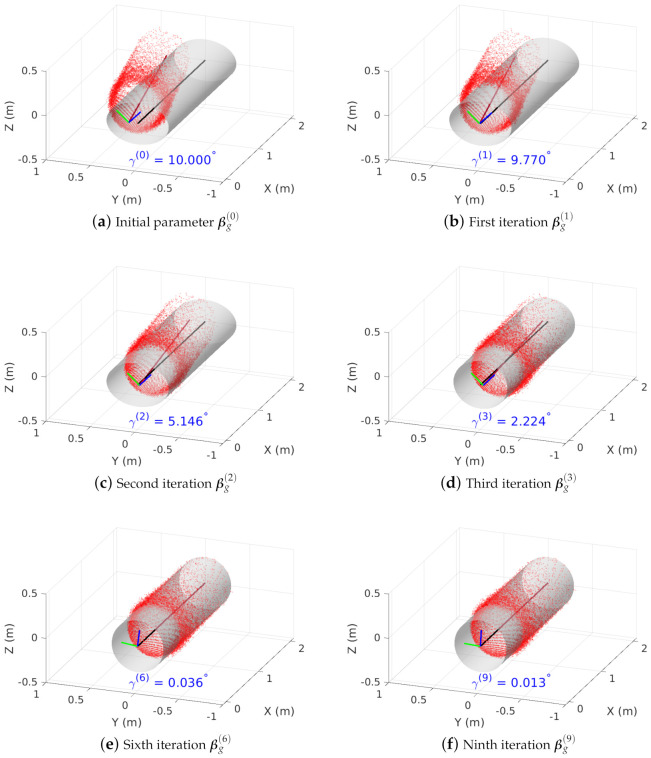
Accuracy of iterative LMA, where the pipe and point cloud are represented by a gray cylinder and *m* red points, respectively.

We first examine the accuracy of the first transformation parameter $\beta_{g}^{\left(k\right)}$ obtained from the iterative LMA. When the OS-0-64 LiDAR is placed at the center of pipe with zero roll, pitch, and yaw angles, Figure 10 shows the accuracy of LMA solutions depending on the number of iterations *k*: The true inner pipe wall with parameter ${\bar{\beta }}_{g}=\left(0.5885,0.5827,0^{\circ },0^{\circ },0^{\circ },0,0\right)$ and LiDAR point cloud transformed by parameter $\beta_{g}^{\left(k\right)}$ are represented by a gray cylinder and *m* red points, respectively. In addition, the X-, Y-, and Z-axes of LiDAR are depicted by red, green, and blue line segments, respectively. The cylindrical pipe and LiDAR X-axes are also shown by black and brown line segments, respectively. The unit vector for pipe axis in GPF is given by $u_{p}=\left(1,0,0\right)$, whereas the unit vector for LiDAR X- at the *k*-th iteration in GPF is represented by the direction cosine

(17) $$u_{l}^{\left(k\right)}=\left(\cos\phi_{g}^{\left(k\right)},\cos\theta_{g}^{\left(k\right)},\cos\psi_{g}^{\left(k\right)}\right).$$

Then, the angle between the pipe and LiDAR X-axes is given by $\gamma^{\left(k\right)}=\arccos\left(u_{p}\;\cdot \;u_{l}^{\left(k\right)}\right)$.

We intentionally generate a high discrepancy between the true cylindrical pipe and transformed LiDAR point cloud by setting initial parameter $\beta_{g}^{\left(0\right)}$ = (0.7027, 0.4685, $45^{\circ }$, $0^{\circ }$, $10^{\circ }$, −0.1, 0), as shown in Figure 10a. The major difference between the true and initial parameters lies in the major- and minor-axis lengths, roll and yaw angles, and Y-axis displacement. After the first LMA iteration in Figure 10b, the discrepancy in the Y-axis displacement has been significantly reduced to $\Delta y_{g}^{\left(1\right)}=8.618$ mm, while the other initial parameters are almost the same. The second LMA iteration in Figure 10c reduces the pitch angle to $\theta_{g}^{\left(2\right)}=5.146^{\circ }$, and the third LMA iteration in Figure 10d improves the estimation accuracy of both major- and minor-axis lengths: $D_{\max}^{\left(3\right)}=0.6096$ m and $D_{\min}^{\left(3\right)}=0.5362$ m. The point cloud of the transformation by the sixth LMA iteration in Figure 10e almost overlaps with the true cylindrical pipe, where $\beta_{g}^{\left(6\right)}=\left(0.5861,0.5848,6.939^{\circ },0.006429^{\circ },-0.0359^{\circ },0.000681,0.000345\right)$. Finally, the transformation parameters $\beta_{g}^{\left(9\right)}=\left(0.5872,0.5837,3.295^{\circ },-0.00911^{\circ },-0.00919^{\circ },0.000155,-0.00005\right)$ after the ninth LMA iteration is very close to the true parameter ${\bar{\beta }}_{g}$ as shown in Figure 10f, except for a few degree difference in roll angle due to the rotational invariance in Section 3.1. In addition, the iterative LMA is computationally efficient to compute parameter $\beta_{g}^{\left(9\right)}$ in 73 msec (For the details of the computational efficiency of SPPE, please refer to Section 4.4). To summarize, the proposed SPPE can achieve an excellent estimation accuracy with a small number of LMA iterations, and be applicable for real-time ILI applications.

### 4.3. Pipe Attributes and PIG Pose

**Figure 11:**
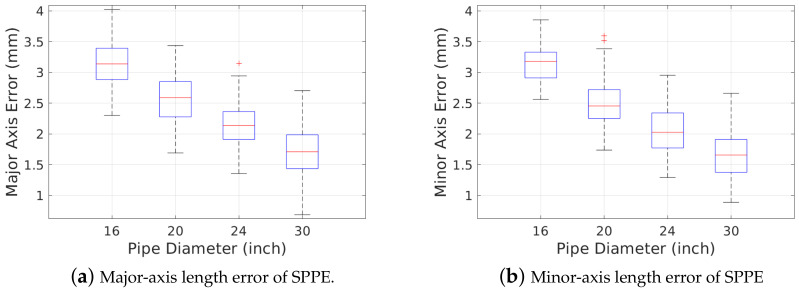
Major- and minor-axis length errors of our SPPE for different pipe diameters.

In this section, we first investigate the accuracy of our SPPE in terms of three pipe attributes: the major- and minor-axis lengths, and ovality angle. Figure 11 shows the estimation error of major- and minor-axis lengths with four different pipe diameters ranging from 16${}^{\prime \prime }$ to 30${}^{\prime \prime }$ when O=1%. We observe that our SPPE slightly overestimates both major- and minor-axis lengths by a few millimeters, but this error decreases with increasing reference pipe diameter. We notice that the worst case diameter estimation error of our SPPE is still less than 4 mm, which is less than 1.0% of the smallest pipe diameter (16${}^{\prime \prime }$).

**Figure 12:**
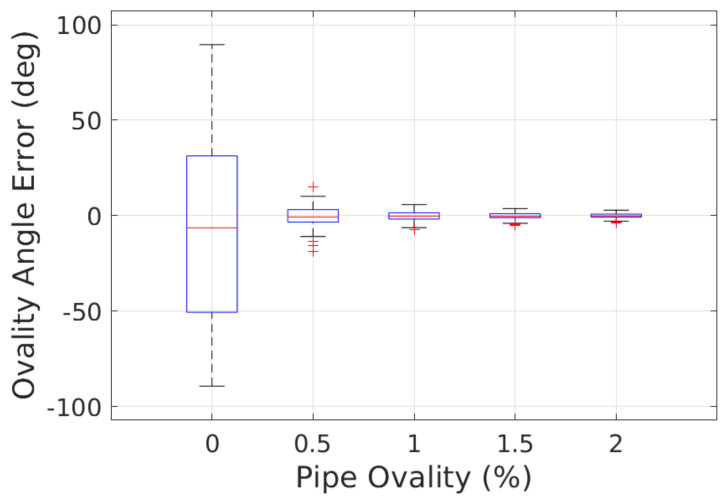
Ovality angle error of our SPPE for different pipe ovalities.

Figure 12 shows the accuracy of ovality angle $\phi_{oval}$ estimation by our SPPE when the reference pipe diameter is *D* = 24". The fact that a LiDAR sensor is oblivious to the rotation in roll angle in a cylindrical pipe with perfectly round cross section (O=0%) incurs a very high uncertainty in the ovality angle estimation. For example, the standard deviation of ovality angle error is given by $51.98^{\circ }$ for O=0%. However, we can see that this variance decreases with the increase of pipe ovality: the standard deviation of the ovality angle error is reduced to $5.675^{\circ }$ when O=0.5%. It is also worth noting that the bias of the ovality angle estimation by our SPPE is very low—less than $0.5^{\circ }$ when O≥0.5%.

**Figure 13:**
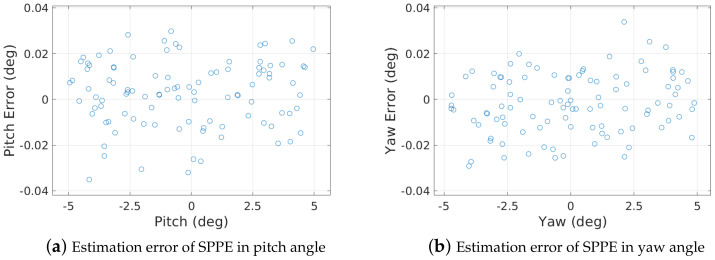
Estimation errors of SPPE in pitch and yaw angles.

Next, we discuss the rotational and translational pose accuracy of our SPPE, including the pitch and yaw angles, and 2D cross sectional displacement, in a 24${}^{\prime \prime }$ pipe with O=1.0%. Figure 13 shows the estimation accuracy of our SPPE in pitch and yaw angles. Considering the LiDAR depth errors in Figure 8, it is surprising that our SPPE can achieve very high estimation accuracy of a rotational PIG pose—the estimation errors for both the pitch and yaw angles are less than $0.04^{\circ }$ regardless of the PIG pose.

**Figure 14:**
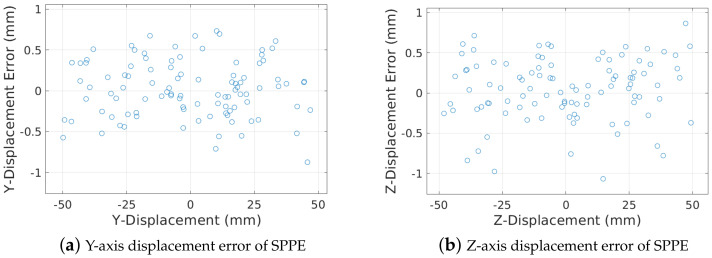
2-D deviation errors of SPPE.

Figure 14 also shows the estimation accuracy of SPPE for the 2D deviation from the center of the pipe in the cross section plane. We observe that our SPPE achieves sub-millimeter-level uncertainty in the estimation of 2D deviation in most cases, regardless of the deviation from the center of elliptical cross section. To conclude, our SPPE approach achieves an outstanding performance in the estimation accuracy of pipe attributes and PIG pose.

### 4.4. Robustness to LiDAR Depth Error and LMA Input Size

**Figure 15:**
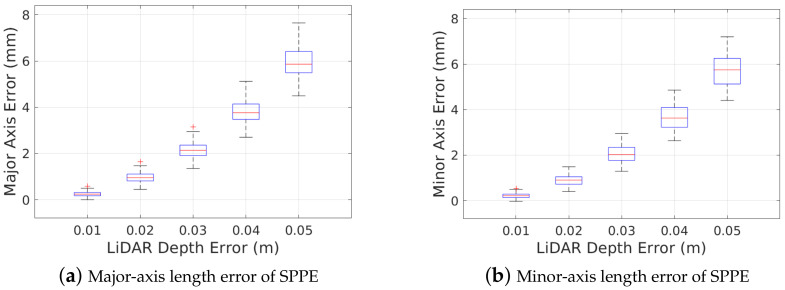
Robustness of SPPE to LiDAR depth error.

In this section, we first discuss the estimation accuracy of our SPPE depending on the LiDAR depth error. Figure 15 shows the accuracy of our SPPE in the estimation of major- and minor-axis lengths for different LiDAR depth errors in a 24${}^{\prime \prime }$ pipe with O=1%. We observe that the uncertainty of LiDAR depth error degrades the estimation accuracy of pipe attributes, which leads to an overestimation of the pipe diameters. Solving the non-linear equation in (4), our SPPE approach can reduce the estimation error of the pipe diameter to approximately one tenth of the LiDAR depth error. Contrary to the estimation of pipe attributes, it can be seen that our SPPE still achieves high accuracy in the estimation of the PIG pose, which is similar to the results in Figure 13 and Figure 14.

Next, we investigate the performance of our SPPE depending on the LMA input size, which must be at least seven to obtain a feasible solution to transformation parameter $\beta_{g}^{*}$. Table 2 shows the mean and standard deviation of estimation errors for pipe attributes and PIG pose by our SPPE approach for a different number of randomly chosen LMA input points in a 24${}^{\prime \prime }$ pipe with O=1%. When the LMA input size is m=10, we observe that the proposed SPPE poorly estimates the pipe diameters and the 2D deviation of PIG pose: first, the overestimation of the pipe diameters by our SPPE can be as high as a few centimeters. Second, it suffers from a high uncertainty of the pipe diameters and 2D deviation. It is also seen that the estimation accuracy of our SPPE improves with the increase of LMA input size *m*: Except for the ovality angle error, which gradually decreases with the number of LMA input points (due to the rotational invariance), the estimation accuracy of our SPPE with LMA input size $m=10^{2}$ achieves almost the same performance as that with $m=10^{4}$.

**Figure 16:**
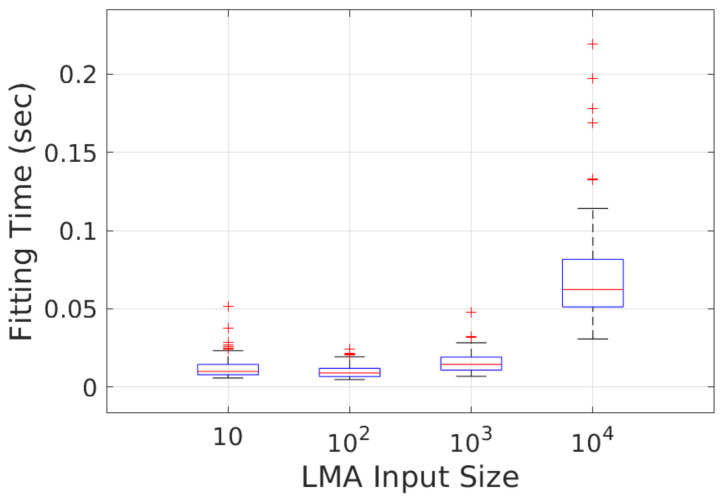
Robustness of SPPE to LMA input size.

Figure 16 shows the computation time of our SPPE on the PC with CPU core i7-10750H (2.6 GHz) and 32 GB RAM. Surprisingly, the computation time of our SPPE with $m=10^{2}$ is the lowest (10.2 msec) among four different LMA input sizes, which is even lower than our SPPE with m=10: 12.3 msec. This is because, for very small *m*, the LMA takes a longer time to converge to an optimal point due to its large searching area for the solution in the parametric space. The computation time of our SPPE is also scalable to a large LMA input size so that it can be a promising solution to real-time estimation of pipe attributes and PIG pose.

## 5. Conclusions

This paper has presented a novel SPPE approach to the pipe-attribute and PIG-pose estimation based on 3D point clouds. We formulate this problem into an optimal transformation matrix estimation problem from a point cloud in the PSF to a point cloud in the global NPF. The basic idea of our SPPE is to decompose this transformation into two sub-transformations: the first one from PSF to GPF, and the second one from GPF to NPF. The extensive simulation results from our PIG simulator demonstrate that our SPPE approach can achieve millimeter-level accuracy for pipe-diameter estimation, sub-degree-level accuracy for PIG-pose estimation, and sub-millimeter-level accuracy for displacement estimation.

## Figures and Tables

**Table 1:** Simulation parameters.

Category	Parameter	Notation	Value	Unit
Pipe attribute	Pipe diameter	*D*	16, 20, 24, 30	inch
	Pipe thickness	*T*	12	mm
	Ovality angle	$\phi_{oval}$	[−180, 180)	${}^{\circ }$
PIG pose	Roll angle	$\phi_{p}$	[−180,180)	${}^{\circ }$
	Pitch angle	$\theta_{p}$	[−5,5]	${}^{\circ }$
	Yaw angle	$\psi_{p}$	[−5,5]	${}^{\circ }$
	Y-axis displacement	$\Delta y_{p}$	[−50,50]	mm
	Z-axis displacement	$\Delta z_{p}$	[−50,50]	mm
LiDAR Specification	Angular resolution	-	2048×64	-
	Depth error	σ	0.03	m
	Field of view	FoV	60	${}^{\circ }$
	Maximum ranging	$x_{\max}$	6	m

**Table 2:** SPPE estimation results for different LMA input sizes.

SPPE Error Metric		LMA Input Size			
		10	$10^{2}$	$10^{3}$	$10^{4}$
Major-axis length (mm)	Mean	27.628	3.874	2.310	2.144
	Std. Dev.	32.637	3.845	1.114	0.383
Minor-axis length (mm)	Mean	−16.673	0.232	1.866	2.059
	Std. Dev.	24.256	3.270	1.053	0.406
Ovality angle (deg)	Mean	−0.822	−1.296	1.965	−0.262
	Std. Dev.	53.093	31.798	9.307	3.093
Pitch angle (deg)	Mean	−0.138	−0.010	0.001	0.003
	Std. Dev.	2.342	0.090	0.026	0.015
Yaw angle (deg)	Mean	0.141	−0.012	0.001	−0.002
	Std. Dev.	2.379	0.096	0.025	0.013
$Y_{p}$-axis displacement (mm)	Mean	−0.313	0.390	−0.033	0.030
	Std. Dev.	34.775	2.893	0.829	0.348
$Z_{p}$-axis displacement (mm)	Mean	−2.099	−0.261	0.032	0.047
	Std. Dev.	32.449	2.804	0.836	0.398
